# Supplementary material for: Shared and modality-specific brain regions that mediate auditory and visual word comprehension
Source: eLife. 2020 Aug 24;9:e56972. doi: 10.7554/eLife.56972 (PMC7470824; doi:10.7554/eLife.56972)
Supplement: Supplementary file 1. — Note that adjectives were comparable with regard to their positive valence (Scott et al., 2019). [file elife-56972-supp1.docx]

# Supplementary Table

**Supplementary File 1.** Target words used in this study were adjectives and numbers. Note that adjectives were comparable with regard to their positive valence ([G. G. Scott, et al., 2019](#_ENREF_95)).

| **Adjectives** | **Numbers** |
| --- | --- |
| Beautiful | Eleven |
| Amazing | Twenty-one |
| Exciting | Thirty-two |
| Fantastic | Forty-three |
| Outstanding | Fifty-four |
| Colourful | Sixty-five |
| Impressive | Seventy-six |
| Glorious | Eighty-seven |
| Meaningful | Ninety-eight |
